# Supplementary material for: Control of biosilica morphology and mechanical performance by the conserved diatom gene Silicanin-1
Source: Commun Biol. 2019 Jun 28;2:245. doi: 10.1038/s42003-019-0436-0 (PMC6599040; doi:10.1038/s42003-019-0436-0)
Supplement: Supplementary file 1 — Supplementary Information [file 42003_2019_436_MOESM1_ESM.docx]

**Supplementary Figures**


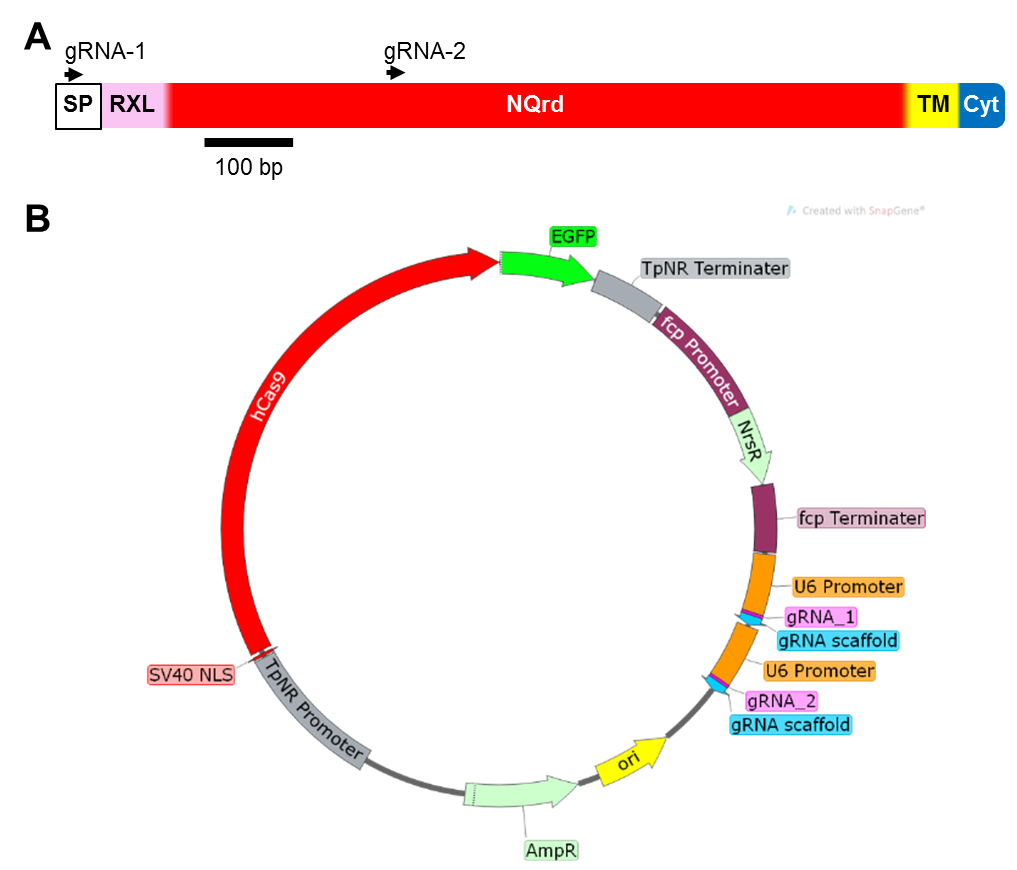


**Supplementary** Figure 1. Design of the plasmid for the Sin1 knockout. (A) Schematic representation of the Sin1 gene (1278 bp) showing the binding sites of gRNA-1 and gRNA-2. Characteristic sequence regions within the Sin1 protein are indicated: SP = signal peptide, RXL = pro-peptide, NQrd = N- and Q-rich domain, TM = transmembrane helix, Cyt = cytosolic domain. (B) Map of plasmid pSin1KO. EGFP = enhanced green fluorescent protein, TpNR = T. pseudonana nitrate reductase, fcp = fucoxanthin chl a/c binding protein, NrsR = nourseothricin acetyltransferase, ori = origin of replication, AmpR = ampicilin resistence gene β-lactamase, SV40 NLS = simian virus 40 nuclear localization signal, hCas9 = human codon optimized CRISPR-associated 9.


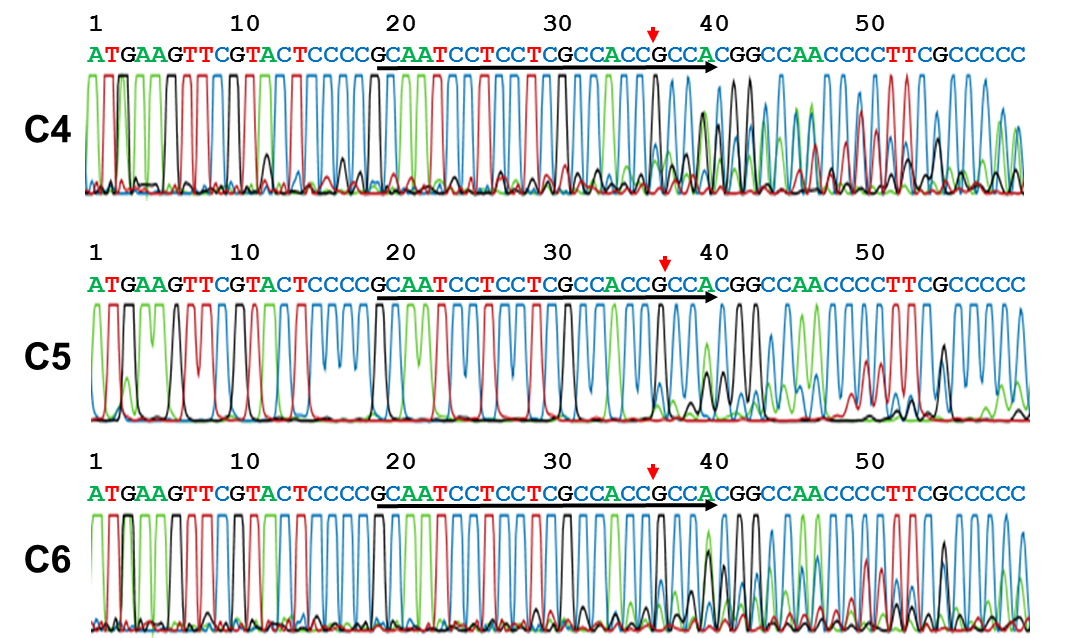


**Supplementary Figure 2. DNA sequences of the products from Sin1-specific genomic PCR from the primary clones C4, C5, and C6 after induction of Cas9 expression.** The Sin1 wild type sequence is shown above the sequencing trace. The binding sites for the gRNAs are indicated by a horizontal black arrow. The vertical red arrows point to the predicted target sites for the Cas9-induced double stand break.

***
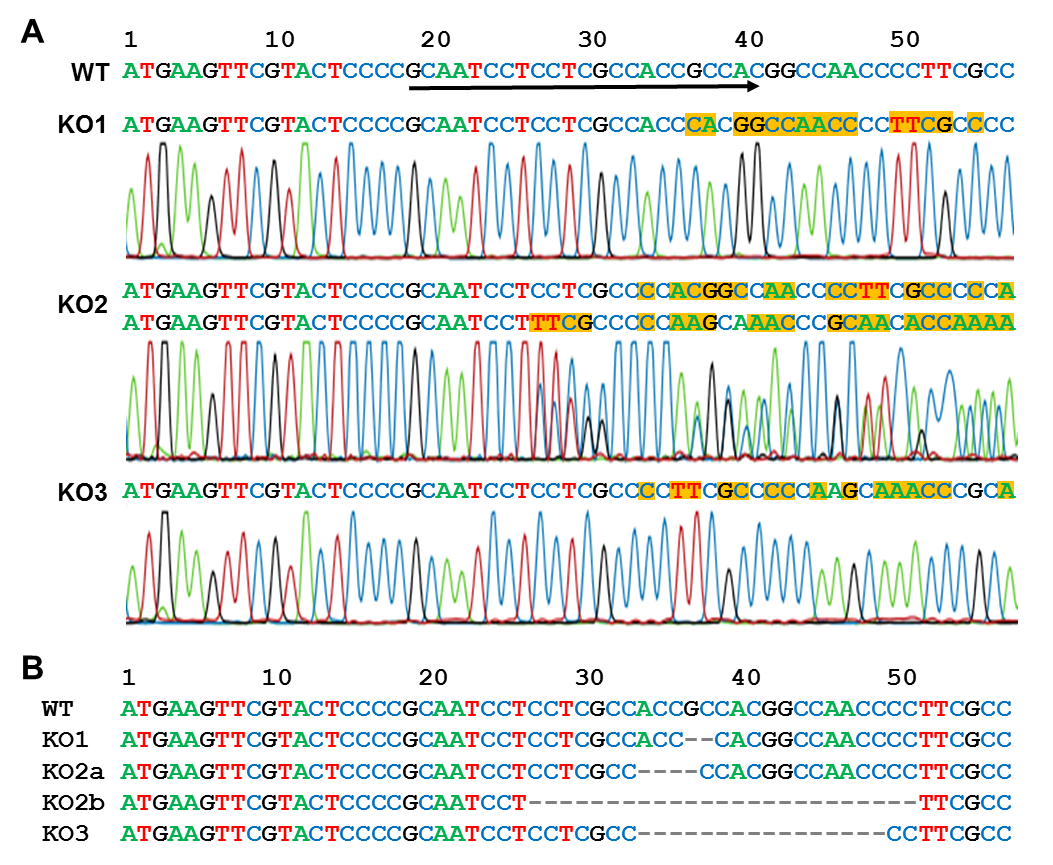
***

**Supplementary Figure 3. Sequence analysis of the Sin1 specific PCR products from three second generation subclones in the region targeted by gRNA-1.** Knockout-1 (KO1) = C4-15-1, knockout-2 (KO2) = C5-21-8, knockout-3 (KO3) = C6-6-1. (A) The top row shows the 5’-region of the *Sin1* wild type gene. The binding site for gRNA-1 is underlined by an arrow. The diagrams labeled KO1 (knockout-1), KO2 (knockout-2) and knockout-3 (KO3) show the sequencing traces of the three second generation sub-clones, and the deduced DNA sequences above each trace. Differences to the wild type *Sin1* sequence are highlighted in orange. The *Sin1* specific PCR product from genomic DNA of *T. pseudonana* sub-clone KO2 (knockout-2) was introduced into *E. coli*, and the plasmids from six *E. coli* clones were sequenced. Each of the *E. coli* plasmids yielded either sequence KO2a or KO2b confirming that *T. pseudonana* sub-clone knockout-2 carries two differently mutated *Sin1* alleles. (B) Comparison of the mutated *Sin1* sequences from (A) with the *Sin1* wild type sequence.

**
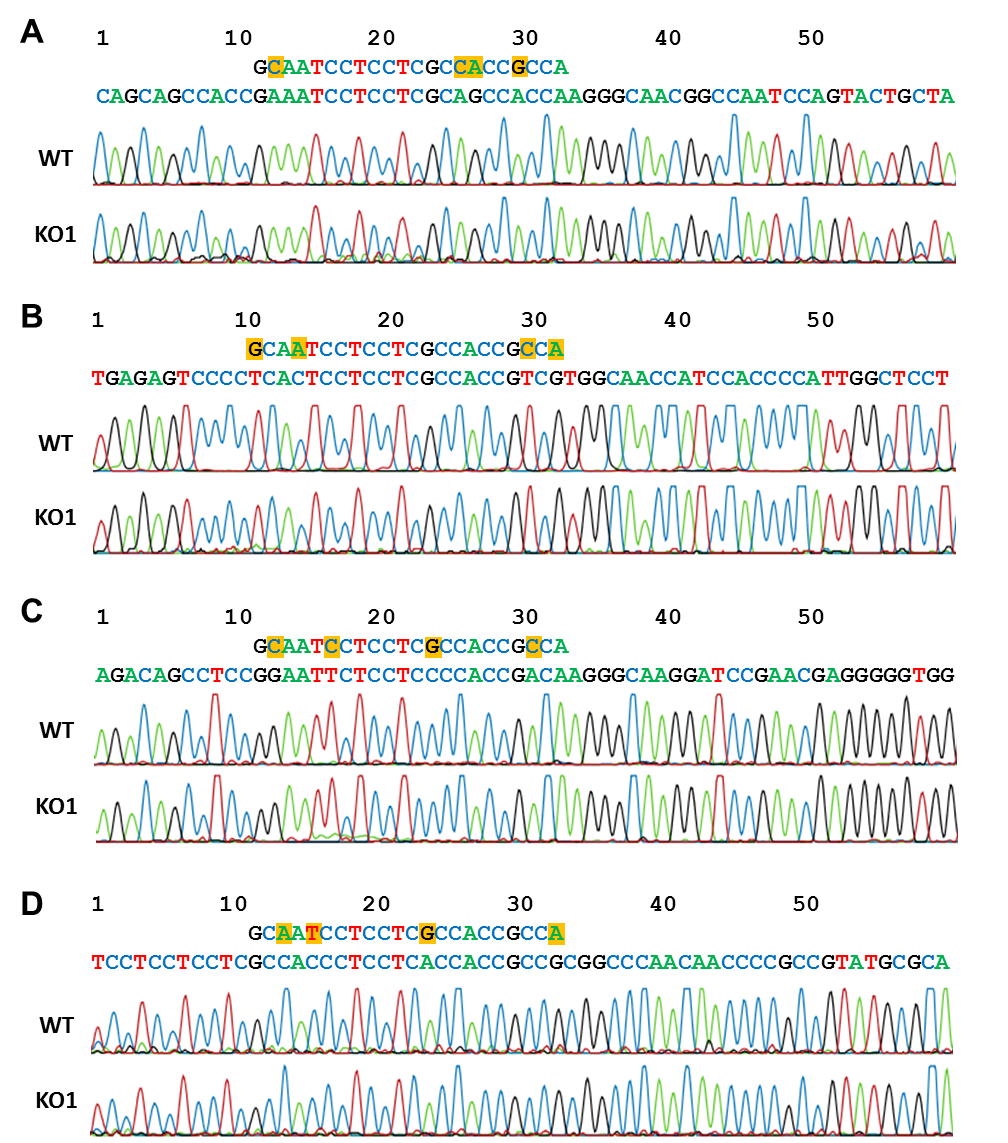
**

**Supplementary Figure 4. Sequence analysis around the potential off-target sites for gRNA‑1 with four mismatches.** Each panel shows on top the nucleotide sequence of gRNA-1 and below the nucleotide sequence of the potential off-target site. Mismatches to the potential off-target site are highlighted in orange in the gRNA-1 sequence. The top trace in each box shows the result from sequencing the genomic PCR product obtained for wild type. The bottom trace shows the sequencing result of the genomic PCR product obtained from mutant knockout-1. (A) Off-target 1, (B) Off-target 2, (C) Off-target 3 und (D) Off-target 4.


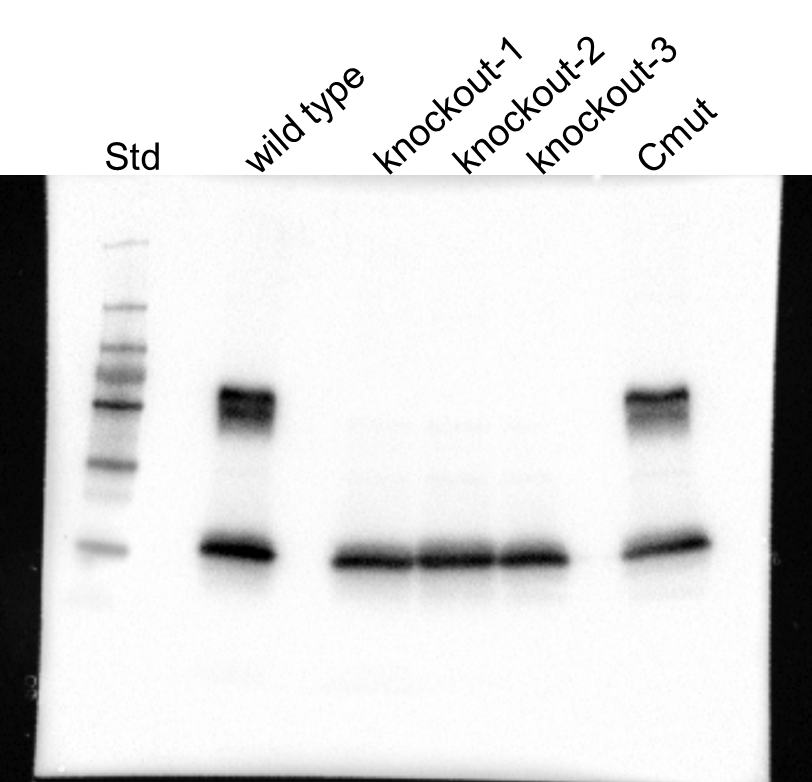


**Supplementary Figure 5. Uncropped image of Western blot for the presence of the Sin1 protein in *T. pseudonana* wild type and mutant cell lines.** Cmut is a cell line that contains an in-frame 3 bp deletion in the *Sin1* gene. The resulting mutated Sin1 protein lacks only one amino acid and thus is expected to run on SDS-PAGE identical to the wild type Sin1 protein. Total lysates from equal amounts of cells were loaded in each lane. The blot was simultaneously probed with anti-Sin1 antiserum^18^ and anti-Hitstone-3 antibodies. Lane Std was loaded with standard proteins of the indicated molecular masses.

| **A** | ****** |
| --- | --- |
| **B** | ****** |
| **C** | ****** |

**Supplementary Figure 6. Growth curves of three independent biological replicates (A-C) of *T. pseudonana* wild type (black), knockout-1 (red), knockout-2 (green) and knockout-3 (blue).** The error bars represent the standard deviation of at least two cell density measurements per data point.

**
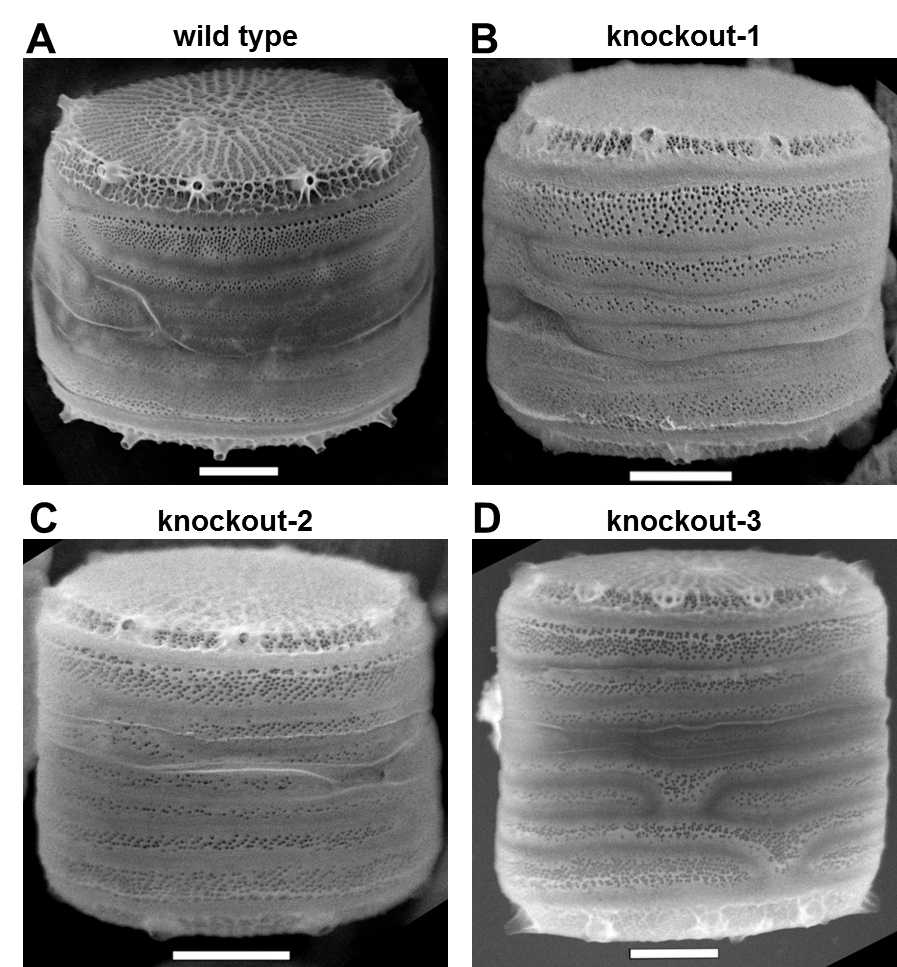
**

**Supplementary Figure 7. SEM images of biosilica in girdle band view.** (A) T. pseudonana wild type, and (B-D) the three Sin1 knockout clones. Scale bars: 1 µm.


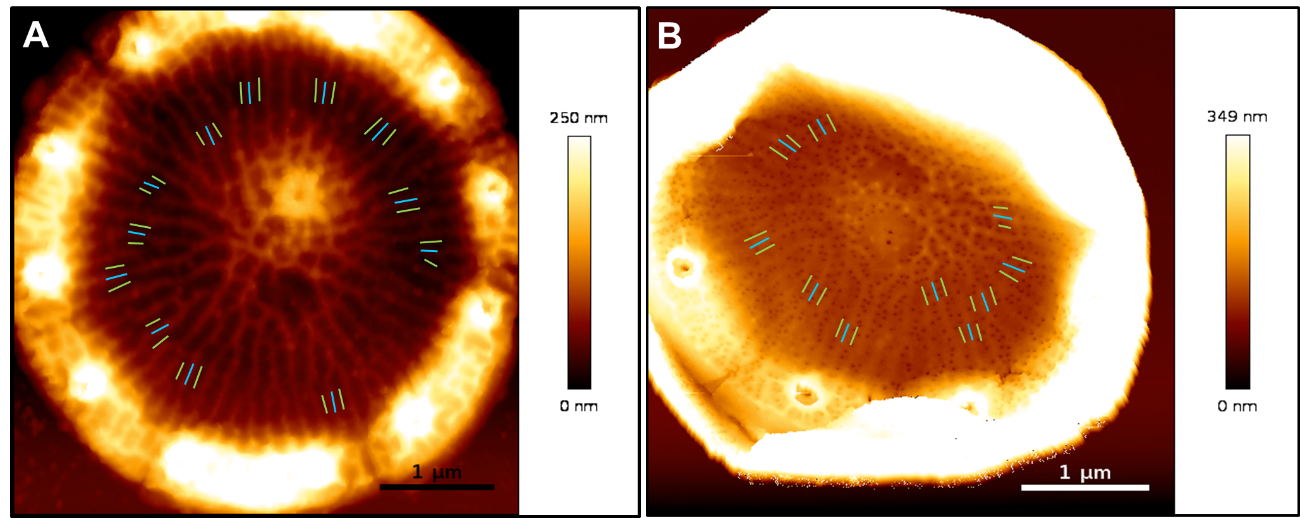


**Supplementary Figure 8. Atomic force microscopy imaging of biosilica valves.** The colored lines indicate some of the segments that were used to measure the z-height of costae. A blue line indicates a costa and a green line indicates the position of a cribrum plate in the middle between two adjacent costae. Representative biosilica valves from (A) wild type and (B) mutant knockout-1 are shown.


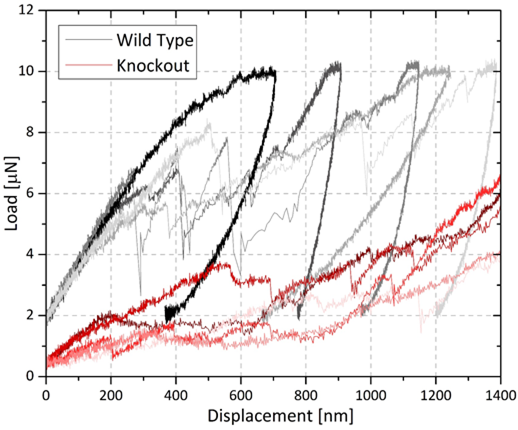


**Supplementary Figure 9. Load displacement curves obtained from load-controlled nanoindentation experiments.** (A) Biosilica cell walls from wild type (grey and black curves), and (B) biosilica cell walls from mutant knockout-1 (red curves). Five cell walls were tested for each strain. It is important to note that at displacements larger than 1 µm the substrate has a pronounced effect on the measured mechanical properties, which results in an increase in the recorded load but does not represent the mechanical performance of biosilica alone.


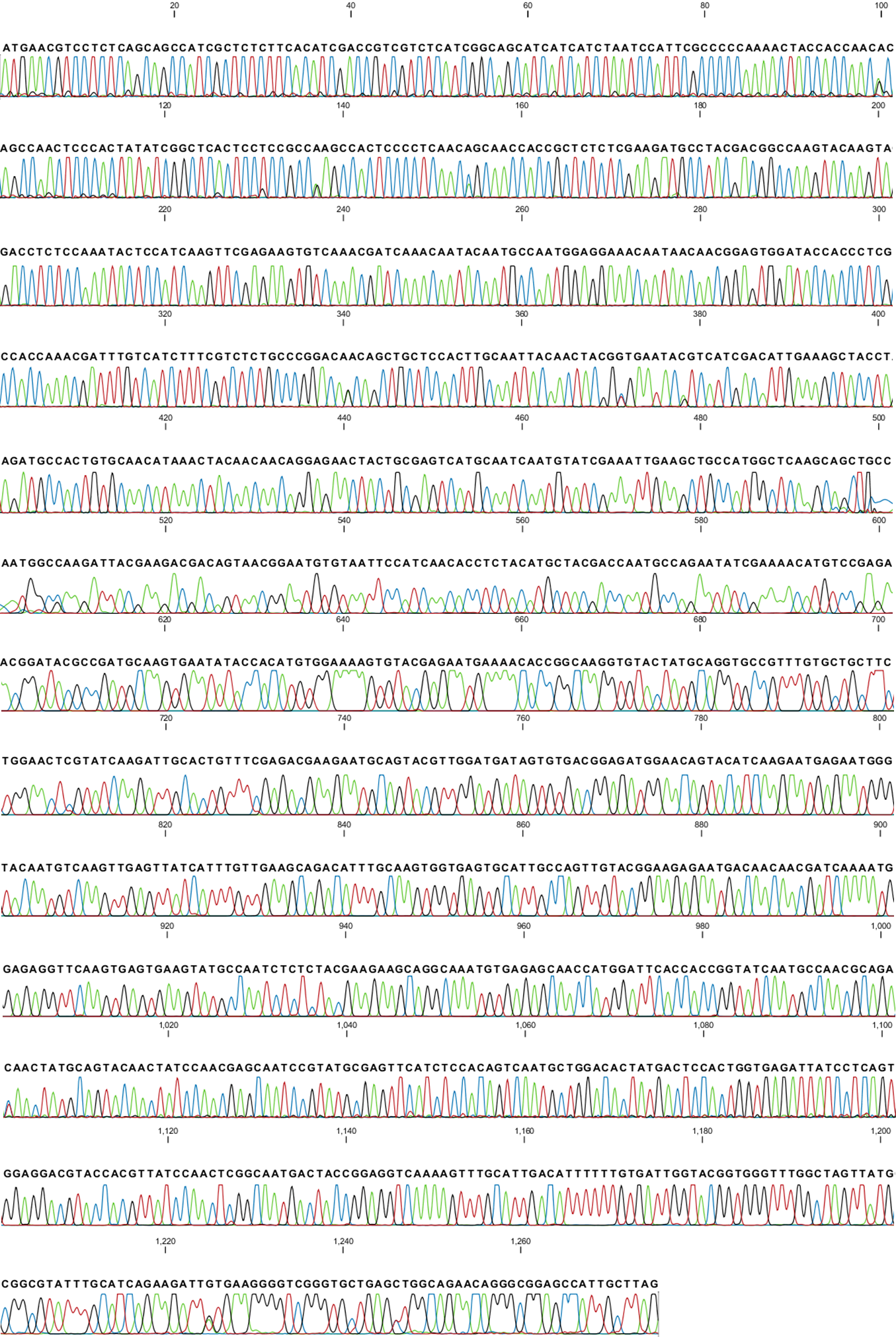


**Supplementary Figure 10. Sequence analysis of the Sin2 coding sequence in *T. pseudonana* mutant knockout-1.** Note that two bases are present at some positions due to allelic variation of the *Sin2* gene.

**TABLES**

**Supplementary Table 1. Off-target prediction for up to five mismatches.**

|  | **Number of targets** | |
| --- | --- | --- |
| **Mismatches** | **gRNA-1** | **gRNA-2** |
| 0 | 1 | 1 |
| 1-3 | 0 | 0 |
| 4 | 4 | 0 |
| 5 | 4 | 2 |

Note that the number of targets for any given number of mismatches does not include the *Sin1* gene.

**Supplementary Table 2. Cell sizes, cell wall surface areas, and silica content of *T. pseudonana* wild type and knockout mutants.**

| **Strain** | **Diameter**  (µm) | **n** | **Length**  (µm) | **n** | **Area**  (µm²) | **Silica per cell**  (fmol) | **Silica density**  (fmol µm^-^²) |
| --- | --- | --- | --- | --- | --- | --- | --- |
| wild type | 5.1 ± 0.5 | 36 | 5.6 ± 0.9 | 87 | 131.6 | 110.7 ± 12.9 | 0.84 |
| knockout-1 | 5.0 ± 0.4 | 27 | 5.8 ± 0.9 | 67 | 129.0 | 72.0 ± 11.0 | 0.56 |
| knockout-2 | 4.9 ± 0.5 | 22 | 5.5 ± 0.8 | 76 | 123.4 | 75.8 ± 6.6 | 0.61 |
| knockout-3 | 4.7 ± 0.4 | 23 | 5.2 ± 1.0 | 77 | 113.1 | 74.0 ± 6.9 | 0.65 |

The silica content and cell size were determined from cells in the linear growth regime at around 10^6^ cells ml^-1^ of the culture. n = number of cells that were measured. Errors represent the standard deviation.
