# Supplementary material for: Control of biosilica morphology and mechanical performance by the conserved diatom gene Silicanin-1
Source: Commun Biol. 2019 Jun 28;2:245. doi: 10.1038/s42003-019-0436-0 (PMC6599040; doi:10.1038/s42003-019-0436-0)
Supplement: Supplementary file 2 — Description of Additional Supplementary Files [file 42003_2019_436_MOESM2_ESM.pdf]

Description of Additional Supplementary Files

File Name: Supplementary Data 1
Description: Source data for Figure 3A

File Name: Supplementary Data 2
Description: Source data for Figure 3B

File Name: Supplementary Data 3
Description: Source data for Figure 4G and Supplementary Figure 9

File Name: Supplementary Data 4
Description: Source data for Table 1 (growth rate) and Supplementary Figure 6

File Name: Supplementary Data 5
Description: Source data for Table 1 (silica content) and Supplementary Table 2

File Name: Supplementary Movie 1
Description: A representative video recorded during a displacement controlled nanoindentation of a single cell wall from T. pseudonana wild type performed inside an SEM.

File Name: Supplementary Movie 2
Description: A representative video recorded during a displacement controlled nanoindentation of a single cell wall from T. pseudonana mutant knockout-1 performed inside an SEM.
