# Supplementary material for: Control of biosilica morphology and mechanical performance by the conserved diatom gene Silicanin-1
Source: Commun Biol. 2019 Jun 28;2:245. doi: 10.1038/s42003-019-0436-0 (PMC6599040; doi:10.1038/s42003-019-0436-0)
Supplement: Supplementary file 8 — Reporting Summary [file 42003_2019_436_MOESM8_ESM.pdf]

nature research

Corresponding author(s): Nils Kröger

Last updated by author(s): Apr 9, 2019

Reporting Summary

Nature Research wishes to improve the reproducibility of the work that we publish. This form provides structure for consistency and transparency in reporting. For further information on Nature Research policies, see Authors & Referees and the Editorial Policy Checklist.

Statistics

For all statistical analyses, confirm that the following items are present in the figure legend, table legend, main text, or Methods section.

n/a	Confirmed	
☐	☒	The exact sample size (*n*) for each experimental group/condition, given as a discrete number and unit of measurement
☐	☒	A statement on whether measurements were taken from distinct samples or whether the same sample was measured repeatedly
☒	☐	The statistical test(s) used AND whether they are one- or two-sided<br>*Only common tests should be described solely by name; describe more complex techniques in the Methods section.*
☒	☐	A description of all covariates tested
☐	☒	A description of any assumptions or corrections, such as tests of normality and adjustment for multiple comparisons
☐	☒	A full description of the statistical parameters including central tendency (e.g. means) or other basic estimates (e.g. regression coefficient) AND variation (e.g. standard deviation) or associated estimates of uncertainty (e.g. confidence intervals)
☒	☐	For null hypothesis testing, the test statistic (e.g. *F*, *t*, *r*) with confidence intervals, effect sizes, degrees of freedom and *P* value noted<br>*Give P values as exact values whenever suitable.*
☒	☐	For Bayesian analysis, information on the choice of priors and Markov chain Monte Carlo settings
☒	☐	For hierarchical and complex designs, identification of the appropriate level for tests and full reporting of outcomes
☒	☐	Estimates of effect sizes (e.g. Cohen's *d*, Pearson's *r*), indicating how they were calculated

Our web collection on statistics for biologists contains articles on many of the points above.

Software and code

Policy information about availability of computer code

Data collection	Data were collected using onboard software, e.g. TriboScan 10 of the in-situ picoindentation system PI 85 (Bruker)
Data analysis	Data were analysed using OriginPro 8 and ImageJ 1.52h

For manuscripts utilizing custom algorithms or software that are central to the research but not yet described in published literature, software must be made available to editors/reviewers. We strongly encourage code deposition in a community repository (e.g. GitHub). See the Nature Research guidelines for submitting code & software for further information.

Data

Policy information about availability of data

All manuscripts must include a data availability statement. This statement should provide the following information, where applicable:

- Accession codes, unique identifiers, or web links for publicly available datasets
- A list of figures that have associated raw data
- A description of any restrictions on data availability

The authors declare that all data supporting the findings of this study are available within the article and its Supplementary Information files or are available from the corresponding author upon request.

Field-specific reporting

Please select the one below that is the best fit for your research. If you are not sure, read the appropriate sections before making your selection.

☒ Life sciences ☐ Behavioural & social sciences ☐ Ecological, evolutionary & environmental sciences

Life sciences study design

All studies must disclose on these points even when the disclosure is negative.

Sample size	For counting cross-connection per image, 20 TEM images were chosen with no girdlebands partially covering the valve to allow clear estimation of cross-connection within the image. To increase confidence, three independent knockout clones were analyzed. For costae z-height estimation, at least 120 data points were analyzed to provide statistical confidence. For nanoindentation experiments, 5 isolated cell walls of Tp wt and KO1 were analyzed, due to the significant and reproducible difference in mechanical behavior.
Data exclusions	TEM images with girdlebands partially covering the valve were excluded from the analysis.
Replication	Counting of cross-connections per image as well as costae z-height estimation was not replicated
Randomization	Randomization was not relevant to this study, due to the presence of three independent knockout clones that were compared to the wild type
Blinding	Blinding was not relevant to our study, due to the presence of three independent knockout clones that were compared to the wild type

Reporting for specific materials, systems and methods

We require information from authors about some types of materials, experimental systems and methods used in many studies. Here, indicate whether each material, system or method listed is relevant to your study. If you are not sure if a list item applies to your research, read the appropriate section before selecting a response.

Materials & experimental systems

n/a	Involved in the study	
☐	☒	Antibodies
☐	☒	Eukaryotic cell lines
☒	☐	Palaeontology
☒	☐	Animals and other organisms
☒	☐	Human research participants
☒	☐	Clinical data

Methods

n/a	Involved in the study	
☒	☐	ChIP-seq
☒	☐	Flow cytometry
☒	☐	MRI-based neuroimaging

Antibodies

Antibodies used	polyclonal anti-Sin1 antiserum rom rabbit
Validation	The antiserum was first described and validated in Kotzsch et al . BMC Biol. 15, 65 (2017).

Eukaryotic cell lines

Policy information about cell lines

Cell line source(s)	T. pseudonana (Hustedt) Hasle et Heimdal clone CCMP 1335
Authentication	A single cell isolate was originally collected from Moriches Bay, Long Island Sound, New York in 1958, and has been maintained continuously in culture since then as CCMP 1335 (Provasoli-Guillard National Center for Culture of Marine Phytoplankton; http://ccmp.bigelow.org/)
Mycoplasma contamination	Cell line was not tested for mycoplasma contamination
Commonly misidentified lines (See ICLAC register)	n/a
